# Multifaceted Interactions Between Endophytes and Plant: Developments and Prospects

**DOI:** 10.3389/fmicb.2018.02732

**Published:** 2018-11-15

**Authors:** Ekta Khare, Jitendra Mishra, Naveen Kumar Arora

**Affiliations:** ^1^Department of Microbiology, Institute of Biosciences and Biotechnology, Chhatrapati Shahu Ji Maharaj University, Kanpur, India; ^2^Department of Microbiology, Babasaheb Bhimrao Ambedkar University, Lucknow, India; ^3^Department of Environmental Science, Babasaheb Bhimrao Ambedkar University, Lucknow, India

**Keywords:** endophytes, biotic stress, abiotic stress, genomics, metabolomics, secondary metabolites

## Abstract

Microbial endophytes are present in all known plant species. The ability to enter and thrive in the plant tissues makes endophytes unique, showing multidimensional interactions within the host plant. Several vital activities of the host plant are known to be influenced by the presence of endophytes. They can promote plant growth, elicit defense response against pathogen attack, and can act as remediators of abiotic stresses. To date, most of the research has been done assuming that the interaction of endophytes with the host plant is similar to the plant growth-promoting (PGP) microbes present in the rhizosphere. However, a new appreciation of the difference of the rhizosphere environment from that of internal plant tissues is gaining attention. It would be interesting to explore the impact of endosymbionts on the host’s gene expression, metabolism, and other physiological aspects essential in conferring resistance against biotic and abiotic stresses. A more intriguing and inexplicable issue with many endophytes that has to be critically evaluated is their ability to produce host metabolites, which can be harnessed on a large scale for potential use in diverse areas. In this review, we discuss the concept of endophytism, looking into the latest insights related to the multifarious interactions beneficial for the host plant and exploring the importance of these associations in agriculture and the environment and in other vital aspects such as human health.

## Introduction

The term endophyte was first introduced by De Bary (1866), defined as any organism that grows within plant tissues, but now they are more precisely described in terms of their types (fungal and bacterial) and relationships (obligate or facultative with the host plant (Petrini, 1991; Cabral et al., 1993; Hallmann et al., 1997; Rosenblueth and Martínez-Romero, 2006). According to Fesel and Zuccaro (2016), a comprehensive definition of endophytes does not specify their functional relationship and apart from commensalistic symbionts, they can exist from latent pathogens or saprotrophs to mutualistic associations. The mutualistic association by colonizing plant tissues both intercellularly and/or intracellularly is a well-versed component of their lifestyle and most of the modern research clearly shows that survival and health of plants are very much dependent upon these microorganisms (Hardoim et al., 2015; Potshangbam et al., 2017). For example, in rhizobia*-*legume symbiosis, which is also considered as one of the best-described endophytic relationships, the bacterial endosymbiont governs plant’s need for nitrogen (Santoyo et al., 2016). The relationship is thought to have evolved 60 million years ago (Sprent, 2008) and, from the beginning, it has played an important role in land ecosystems, providing benefits for both the partners. It has been proposed that endophytes have originated from the rhizosphere microbes or seed-borne microbial communities, but genome studies and their correlation with them show that these microbes are far more versatile and may contain genes for novel traits beneficial to the host plant (Ali et al., 2014). However, we are yet to identify these specialized genes designated specifically for the endophytic lifestyle. In order to sustain stable symbiosis, endophytes manufacture or induce the host plant to produce metabolites that promote plant growth and help them adapt better to the environment (Das and Varma, 2009). Endophytes play an imperative role to maintain the health of plants, as they can protect or prepare the plant against abiotic and biotic stresses and help in enhancing growth and yields (Tanaka et al., 2005; Vega et al., 2008; Lugtenberg et al., 2016; Lata et al., 2018).

**FIGURE 1:**
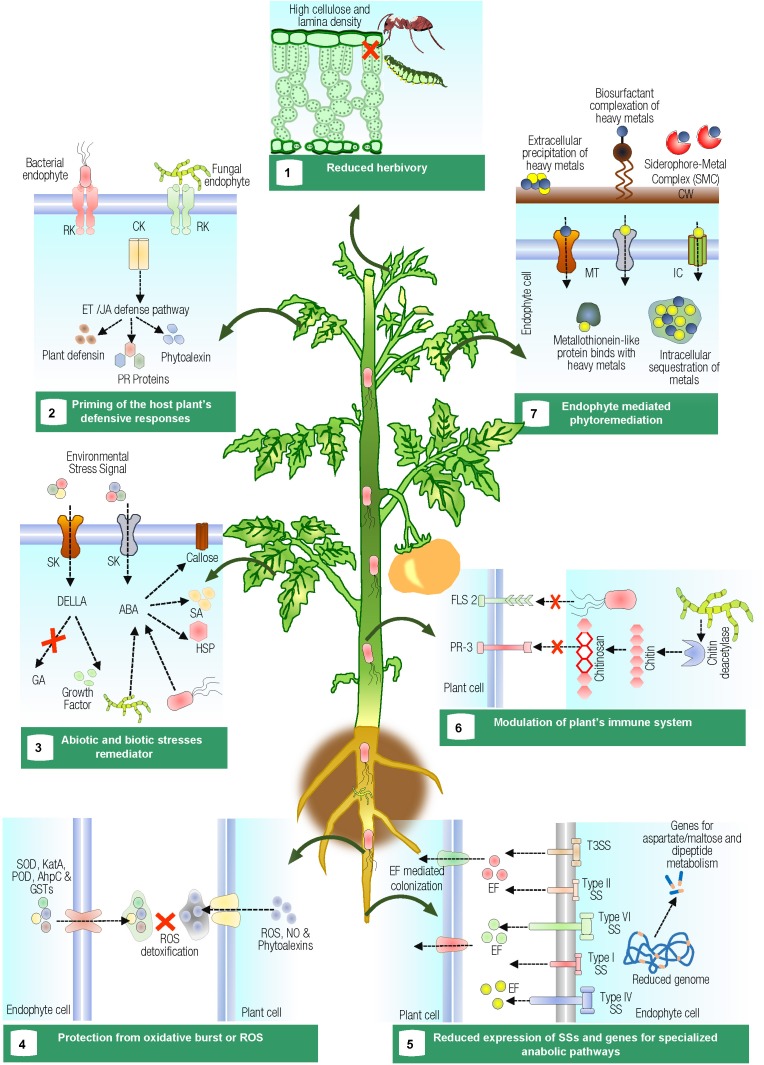
Pictorial representation showing multifaceted interaction of endophytes with host plants. **(1)** Fungal endophytes change chemical and physical characteristics of the leaf such as high-cellulose content and lamina density, which provide toughness resulting in reduced herbivory rates, specifically by leaf–cutting ants. **(2)** Endophytes prime the host plant’s defensive responses against phytopathogens. Early detection of the phytopathogen by cell surface receptor kinases (RK) and subsequent cytoplasmic kinases (CK) mediate intracellular responses and trigger ethylene/jasmonic acid transduction pathway. **(3)** Abiotic and biotic stress (signals positively induce expression of the stress-responsive genes, preinvasion defense, and enhanced callose deposition. However, ABA affects negatively signals that trigger systemic acquired resistance. The endophyte significantly modulate stress through the downregulation of ABA. Gibberellins synthesized by plants or endophytes hamper the inhibitory effects of DELLA proteins over the plant-growing signals. **(4)** Reactive oxygen species (ROS), generated by the plant, are neutralized by the production of enzymes such as superoxide dismutases (SOD), catalases (CatA), peroxidases (POD), alkyl hydroperoxide reductases (AhpC), and glutathione-*S*-transferases (GSTs ) in endophytes. **(5)** Protein secretion systems (SSs), which deliver effector proteins (EF) into the plant are either absent or present in low abundance in mutualistic endophytic bacteria. Endophytes also encode specific genes for utilizing aspartate/maltose and dipeptides metabolism. **(6)** Fungal endophytes modulate the plant’s immune system by the production of chitin deacetylases, which deacetylate chitosan oligomers and, hence, prevent themselves from being recognized by chitin-specific receptors (PR-3) of the plants that recognize chitin oligomers. Perception of flagellin (FLS 2) from endophytes also differs from phytopathogens. **(7)** Endophytic microbes alleviate metal phytotoxicity via extracellular precipitation, intracellular accumulation, sequestration, or biotransformation of toxic metal ions to less toxic or non-toxic forms. Where, RK, receptor kinase; CK, cytoplasmic kinase; ET, ethylene; JA, jasmonic acid; SK, sensor kinase; GA, gibberellic acid; DELLA, DELLA protein; ABA, abscisic acid; SA, salicylic acid; HSP, heat shock protein; ROS, reactive oxygen species; SOD, superoxide dismutases; CatA, catalases; POD, peroxidases; AhpC, alkyl hydroperoxide reductases; GSTs, glutathione-s-transferases; EF, effector protein; PR-3, chitin-specific receptors; FLS 2, flagellin; TTSS, type III secretion system; SS, secretion system; MT, metal transporters; IC, ion channels; CW, bacterial cell wall.)

The emergence of the “plant microbiome” concept has changed the scenario completely and, hence, the coevolution of plants and their symbionts has to be looked upon for determining the factors involved in coexistence of both the partners and tracking the benefits out of the relationship (Turner et al., 2013). Induction of plant genes expression in the presence of endophytes provides clues about their effects on the host plant (Berendsen et al., 2015). The modern “omics”-based approaches including genome sequencing, comparative genomics, microarray, next-generation sequencing (NGS), metagenomics, and metatranscriptomics may provide an in-depth detail on endophytic lifestyle (Kaul et al., 2016). The present review focuses on the multidimensional interactions between endophytes and their plant hosts, particularly, in relation with maintaining the health of the plant. An overview to this approach is given in Figure 1.

## Host Plant Factors and Endophytic Lifestyle

Successful endophyte colonization involves compatible plant-microbe interactions. As the endophyte invades the host plant, it is recognized by the plant, and cross-talk of signal molecules is initiated. Various studies have shown the chemotactic response of endophytes to root exudates of host plants (Rosenblueth and Martínez-Romero, 2006; Compant et al., 2010; Brader et al., 2014). Root exudates are rich in biomolecules, which attract or are recognized by friendly microbes including endophytes. Exudates are also rich in nutrients and water that attract all sorts of microbes. Flavonoids are one such metabolite secreted by several plants and categorized as chemoattractants, which play an important role in endophytic interaction with the root hair. Flavonoids are already being used in bioformulations for effecting successful infection of legume roots by rhizobia (Arora and Mishra, 2016). Flavonoids are also reported to play a role in case of non-rhizobial endophytes, and it was proved that in the presence of these metabolites, the colonization of root in rice and wheat by the endophytic *Serratia* sp. EDA2 and *Azorhizobium caulinodans* ORS571 was far more effective (Webster et al., 1998; Balachandar et al., 2006). Lipo-chitooligosaccharides (LCO), also called the Nod factors, are well-known signal molecules activating the common symbiotic pathway (CSP) in rhizobia-legume and arbuscular mycorrhizal associations (Gough and Cullimore, 2011). Recently, strigolactone (SL) secreted by roots of *Arabidopsis thaliana* was found to act as a signal molecule for colonization of endophytic *Mucor* sp (Rozpa¸dek et al., 2018). According to López-Ráez et al. (2017), SL treatment may also activate synthesis and release of short-chain chitin oligomers, which can stimulate the symbiotic signaling pathway in the plant. Studies also suggest the crucial role of arabinogalactan proteins (AGPs), which are highly glycosylated members of the hydroxyproline-rich glycoprotein (HRGP) superfamily of plant cell wall proteins, in establishing interaction of plant with microbe (including endophyte) at several stages (Nguema-Ona et al., 2013). These proteins have a definite role in root colonization, working as repellents or attractants for microbes and in the development of infection structures (Nguema-Ona et al., 2013). Several other root exudates including sugars, amino acids, organic acids, phenolic compounds, and other secondary metabolites are now known to be secreted by plant roots, which selectively invite the mutualistic microbes, particularly the endophytes (Chagas et al., 2017).

Apart from this, it is still a matter of research to find out the strategies plants employ to distinguish beneficial microbes such as endophytes from pathogens. There is evidence, which suggests the role of the plant innate immune system (discussed later in this review) in allowing beneficial microbes including endophytes to enter plant tissues (Fesel and Zuccaro, 2016). Recent findings on plant gene expression and micro-RNAs (miRNAs) suggest that in plants the response of genes and pathways depend upon the endophytic microbe. However, signaling pathways of ethylene (ET)/jasmonic acid (JA)/salicylic acid (SA) work irrespective of the microbe. Kusajima et al. (2018) found that bacterial endophyte *Azospirillum* sp. B510 induces systemic disease resistance in rice and further, gene expression analysis indicated that ET signaling is required for endophyte-mediated induced systemic resistance (ISR) in rice. Several studies have proved that there is downregulation of plant defense pathways during the colonization of plants by mutualistic partners such as rhizobia or arbuscular mycorrhizal fungi (AMF) (Fouad et al., 2014; Benhiba et al., 2015; Sarkar et al., 2016). However, during mutualistic interactions, late induction of SA/JA/ET signaling pathways prevents the microbe from ‘overstepping’ and ‘overpowering’ the plant (Plett and Martin, 2018). It is reported that the majority of miRNAs induced in the host during the establishment of endophytes also target hormone-response pathways (Formey et al., 2014). During AMF infection, the miRNA – E4D3Z3Y01BW0TQ – is upregulated and disrupts gibberellic acid (GA) signaling pathway, known for repressive action against mutualistic associations (Formey et al., 2014; Martín-Rodríguez et al., 2015; Wu et al., 2016). The plant may also induce expression of different groups of genes during colonization by diverse sets of microbes. For example, during the establishment of symbiosis, the majority of pathways targeted by miRNAs for plant defense system are turned off that would otherwise have obstructed proliferation of endophytes (Plett and Martin, 2018).

The comparative genomic analysis provides insights into endophytic behavior. Hardoim et al. (2015) reported that among endophytes, genes involved in anabolic pathways are more diverse and abundant; however, catabolism-related genes, particularly those that are involved in the invasion of the host, are more prominent among phytopathogens. The coexistence of genes for nitrogenase and ribulose bisphosphate carboxylase/oxygenase (RuBisCO) has worked as a specific marker for endophytes with symbiotic nitrogen fixation abilities (Karpinets et al., 2014). The advancements in mutualistic symbioses research indicate that plants via nutrient monitoring are able to identify whether the invading microbe is beneficial or a parasite (Plett and Martin, 2018).

Lateral gene transfer plays a significant role in promoting genetic diversity and acquiring characteristics important for colonizing the endosphere of plants and production of secondary metabolites, which provide an edge to both the partners (Tisserant et al., 2013; Arora et al., 2018). An example showing a beneficial role of lateral gene transfer is the presence of the gene for mannitol dehydrogenase in several bacterial endophytes, an enzyme involved in defense against phytopathogenic fungi. This gene gives endophytes a competitive advantage in the endosphere (Wu et al., 2011). The endophytic *Enterobacter* sp. is reported to have genes for amino acid/iron transport, hemolysin, and hemagglutinin, which are important for host-bacterium interactions, on a large conjugative plasmid (Taghavi et al., 2010). The location of potent genes can thus give clues about endophytic lifestyle. For further information on endophytic genomic markers, one can refer to works of Shidore et al. (2012), Karpinets et al. (2014), Hardoim et al. (2015), and Xu et al. (2016).

Advances in metagenome studies have revealed important findings regarding the colonization of bacteria in the plant endosphere (Sessitsch et al., 2012). A near-complete genome of endophytic *Verrucomicrobia* showed a reduction in genome size by almost half in comparison with normal soil bacteria (Brewer et al., 2016). Obligate plant endosymbiotic bacteria undergo a reduction in genome size during evolution as an adaptation to dependency on hosts for several activities (Hottes et al., 2013). Diverse bacterial groups of endophytes are known to have a large expansion of insertion sequences (IS). According to Song et al. (2010), IS results in genomic reduction and might be responsible for huge diversity among plant microbiome. However, not a single factor or mechanism can be ascertained for endophytic lifestyle and much more is yet to be discovered.

The species and genotype of host plants also significantly influence plant endosphere microbiome (Rodriguez-Blanco et al., 2015; Ding and Melcher, 2016). In certain endophytes, alteration of their lifestyle to pathogenic state is also found to depend on the host genotype, in addition to locally occurring abiotic stress factors (Bacon et al., 2008). For example, in maize, *Fusarium verticillioides* can live as a pathogen or an endophyte (Oren et al., 2003). Similarly, *Ramularia collo-cygni*, during crop development, lives as an asymptomatic endophyte, but later in the growing season can switch to be necrotrophic pathogen (Walters et al., 2008). However, precise external or endogenous factors responsible for fungal transition from endophyte to pathogen are not fully understood. To understand better the dynamics of endophytism, there is a need to carry out comparative studies that work out conditions and gene expressions (in both plants and endophytes) under which the same microbe behaves as mutualist or pathogen.

## Modulation of Plant’s Immune System by Endophytes

A black box for researchers is to track and find out the mechanisms of how endophytes thrive inside the host. So, to enter the host plant, endophytes have to pass through the first line of defense of the plant immune system. This involves recognition of conserved molecules, characteristic of many microbes, also known as microbe- or pathogen-associated molecular patterns (MAMPs or PAMPs) by plants (Newman et al., 2013). Flagellin (Flg;flg22), elongation factor TU (EF-Tu;elf18/26), peptidoglycan (PGN), lipopolysaccharides (LPS), bacterial cold shock proteins (RNP1motif), bacterial superoxide dismutase (Sod), BetaGlycan (GE), β-glucans from oomycetes, and chitin are the most worked upon MAMPs (Newman et al., 2013). These MAMPs are recognized on the surface of plant cells by pattern recognition receptors (PRRs). In the case of fungal endophytes, chitin-specific receptors (PR-3) on the plants recognize chitin oligomers formed on the fungal cell wall, which, in turn, trigger further defensive reactions (Sanchez-Vallet et al., 2015). However, endophytes also work out mechanisms to protect themselves from plant defense mechanisms. For example, Cord-Landwehr et al. (2016) reported that fungal endophytes produce chitin deacetylases, which deacetylate chitosan oligomers that are not perceived by plants’ receptors; hence, they prevent themselves from being recognized. There is also evidence where endophytic bacteria are known to produce their own MAMPs, which are either not recognized by PRRs of plants or plants trigger a comparatively weak and transient defense reaction compared to pathogenic interactions (Vandenkoornhuyse et al., 2015). Trda et al. (2015) showed that in grapevine, perception of flagellin (FLS 2) from an endophytic *Burkholderia phytofirmans* was different from those of bacterial pathogens such as *Pseudomonas aeruginosa* or *Xanthomonas campestris.* In case of oxidative burst or generation of reactive oxygen species (ROS) as plant defense system, endophytes protect themselves by producing enzymes such as superoxide dismutases (SOD), catalases (CatA), peroxidases (POD), alkyl hydroperoxide reductases (AhpC), and glutathione-S-transferases (GSTs) (Zeidler et al., 2004). Protein secretion systems (SSs) in bacteria also modulate the plant immune system. Among all known SSs, type III secretion system (T3SS) and type IV secretion system (T4SS) are essential for delivering effector proteins (EF) by the pathogenic bacteria into the plant, but these are either absent or present in low abundance in mutualistic endophytic bacteria (Green and Mecsas, 2016; Liu et al., 2017). Notable exceptions can be seen in some rhizobial strains where T3SS is important for nodulation of some legumes (Ausmees et al., 2004; Okazaki et al., 2013, 2016). The T3SS is also a determinant for rice endophyte colonization by non-photosynthetic *Bradyrhizobium* (Piromyou et al., 2015). While in mutualistic proteobacterial endophytes, type VI secretion systems (T6SSs) are present that are commonly found in commensal and pathogenic plant-associated bacteria and associated with important functions, which are apart from virulence, usually such as competition against other bacteria (Reinhold-Hurek and Hurek, 2011; Bernal et al., 2018).

## Endophytes Affecting Host Genetic and Phenotypic Expressions

### Increase in Resistance to Biotic Stresses

Potential of endophytes suppress phytopathogens via antagonistic activity has been known (Miller et al., 2002; Gunatilaka, 2006). Endophytes are now known to play roles in inducing ISR against phytopathogens in plants (Kloepper and Ryu, 2006). Foliar endophytes are also reported to regulate the host genetic expression affecting plant physiological responses and defensive pathways (Van Bael et al., 2012; Estrada et al., 2013; Salam et al., 2017). Salicylic acid and jasmonic acid, in particular, are known to play vital roles during plant stress responses against phytopathogens (Khare et al., 2016). Gibberellin-producing endophytes are known to enhance resistance against the attack of phytopathogens and insects through SA and JA pathways (Waqas et al., 2015). Kavroulakis et al. (2007) reported that *Fusarium solani* elicits ISR against *Septoria lycopersici* (tomato foliar pathogen) via induction of pathogenesis-related genes expression in root tissues. *Theobroma cacao* inoculated with foliar endophytic fungi, *Colletotrichum tropicale*, showed a reduction in *Phytophthora* infection (Mejia et al., 2008). Inoculation of *C. tropicale* resulted in elicitation of many components of the ET defence pathway and several other signaling genes responsible for disease resistance in *T. cacao*, *A. thaliana*, and other host plants (Mejía et al., 2014). Endophytic bacteria are known to produce various volatile organic compounds (VOCs) with broad-spectrum antimicrobial activity against phytopathogenic bacteria, fungi, and nematodes. In a study, Sheoran et al. (2015) reported that black pepper-associated endophytic *Pseudomonas putida* BP25 inhibits various phytopathogens such as *Phytophthora capsici, Pythium myriotylum, Gibberella moniliformis, Rhizoctonia solani, Athelia rolfsii, Colletotrichum gloeosporioides*, and plant parasitic nematode, *Radopholus similis*, by several volatile substances. Arora et al. (2001) reported the role of siderophore-producing *Rhizobium* in biocontrol of phytopathogen *Macrophomina phaseolina* causing charcoal rot in a number of crops. Mercado-Blanco et al. (2004) isolated endophytic *P. fluorescence* from roots of olive trees antagonistic against *Vertcillium*. Etminani and Harighi (2018) for the first time reported antagonistic strains of endophytic *Pantoea, Bacillus, Pseudomonas, Serratia*, and *Stenotrophomonas* from wild pistachio showing control of *Pseudomonas syringae* and *Pseudomonas tolaasii.*

Plants are known to produce low-molecular weight antimicrobial molecules called phytoalexins that include various groups of metabolites like terpenoids (Gao et al., 2010). A study by Yong et al. (2009) showed an enhancement in content of terpenoids and growth of *Euphorbia pekinensis* by endophytic fungi *Fusarium* spp. Comparative studies by several workers showed that endophytes-containing plants report relatively high cellulose content and lamina density, exhibiting high leaf toughness, thus resulting in reduced herbivory rates, specifically by leaf-cutting ants (Van Bael et al., 2012; Estrada et al., 2013). The presence of endophytes in host tissues can thus enhance their resistance against pathogens by eliciting the host response or by producing antagonistic metabolites themselves. Gene pools of endophytes and the host plant thus work in tandem to protect the plant from parasites.

### Increase in Resistance to Abiotic Stresses

Abiotic stresses such as drought, salinity, extreme temperatures, heavy metal toxicity, and oxidative stress are severe threats to agroecosystems (Wang et al., 2003; Khare and Arora, 2015). The molecular mechanisms adopted by endophytes for increasing stress tolerance in plants include induction and expression of stress-responsive genes, generation of scavenger molecules like ROS, and synthesis of antistress metabolites (Lata et al., 2018).

Phytohormones play a critical role in tolerance of abiotic stress in plants (Wani et al., 2016). The plant hormone abscisic acid (ABA)-mediated stomatal closure and plant growth regulation contributes to combat osmotic and other abiotic stresses in the plant (Waqas et al., 2012). The ABA biosynthesis and ABA-mediated signaling pathways get modulated by the presence of beneficial microorganisms in the endosphere of plants, which may contribute to the plant growth enhancement under salt stress conditions. Recently, modulation of an ABA-signaling cascade by halo-tolerant *Dietzia natronolimnaea*, responsible for salinity tolerance in wheat plants, was validated by the upregulation of TaABARE (ABA-responsive gene) and TaOPR1 genes (12-oxophytodienoate reductase 1) (Ilangumaran and Smith, 2017). Peskan-Berghofer et al. (2015) demonstrated the requirement of ABA in the establishment of mutualistic symbiosis between beneficial fungus *Piriformospora indica* and *A. thaliana* roots. The upregulation of aquaporin, dehydrin, and malonialdehyde genes has been reported in mitigating abiotic stresses in rice by inoculation of endophytic *Trichoderma harzianum* (Pandey et al., 2016c).

A study on the potato endophyte, *Burkholderia phytofirmans* PsJN, showed modulated expression of genes for a cell surface signaling element (extracytoplasmatic function group IV sigma factors), which allows bacteria to sense changing environmental conditions and refine their metabolism accordingly (Sheibani-Tezerji et al., 2015). The drought-induced osmotic stress resistance associated with PsJN was evidenced by upregulated transcripts that mainly are involved in transcriptional regulation, cellular homeostasis, and the detoxification of ROS. Stress-related gene expression and metabolite levels increased earlier, faster, and at higher levels in PsJN bacterized grapevine over non-bacterized control at low temperatures by harmonizing carbohydrate metabolism (Fernandez et al., 2012). In a recent study, de Zélicourt et al. (2018) reported that a desert plant endophyte *Enterobacter* sp. SA187 colonizes both the surface and inner tissues of *Arabidopsis* roots and shoots and induces salt stress tolerance by the production of bacterial 2-keto-4-methylthiobutyric acid (KMBA), which modulates the plant ET signaling pathway. This novel mechanism utilized by *Enterobacter* sp. SA187 was found to be effective in enhancing the yield of alfalfa crops under salt stress conditions. Endophytic fungus *Curvularia protuberata* has been associated with the survival of the grass *Dichanthelium lanuginosum* at high soil temperatures, particularly, in the Yellowstone National Park (Márquez et al., 2007).

Studies suggest the role of endophytic bacteria in the reduction of metal phytotoxicity via extracellular precipitation, intracellular accumulation, sequestration, or biotransformation of toxic metal ions to less toxic or non-toxic forms (Ma et al., 2016; Mishra et al., 2017). Heavy metal-induced oxidative damage can also be prevented by endophytes through modulation in the activity of plant antioxidant enzymes and by lipid peroxidation (Wan et al., 2012). Madhaiyan et al. (2007) reported that inoculation of endophytic bacteria *Methylobacterium oryzae* and *Burkholderia* sp. reduced the toxicity and accumulation of Ni and Cd and further translocation from roots to shoots of tomato plants. A wide range of endophytic Proteobacteria have been shown to protect plants from drastic effects of herbicides by contributing to their metabolism (Ngigi et al., 2012). In a study, Germaine et al. (2006) reported that on exposure to 2, 4-dichlorophenoxyacetic acid, plants with *Pseudomonas* endophyte showed no accumulation of the herbicide in their tissues. The genome of *Pseudomonas punonensis* D1-6 reveals many herbicide-resistance and -metabolizing genes that indicate the role of this endophytic bacteria in herbicide resistance in the host (Lafi et al., 2017).

## Metabolomics of Endophytes and Role in the Production of Plant Secondary Metabolites

Over a long period, coexistence and evolution of endophytes along with their host plants have established a special relationship significantly influencing the production of bioactive metabolites in plants (Jia et al., 2016). The communication of endophytic communities with the host plant significantly influences physiological processes of the plant such as activation of silent gene clusters leading to the synthesis of novel secondary metabolites.

Now, it is a well-established fact that both the plant and their endophytes can produce an array of common secondary metabolites from similar precursors. Such bioactive compounds include antibiotics (Martinez-Klimova et al., 2017), antitumor bisindole alkaloids (vinblastine and vincristine) (Kumar et al., 2013), anticancer drug camptothecin (Puri et al., 2005), podophyllotoxin (Puri et al., 2006), and insecticide azadirachtin (Kusari et al., 2012). One of the most-studied bioactive compounds with anticancer activity is the synthesis of taxol from yew plant and from its fungal endophyte *Taxomyces andreanae* (Zhou et al., 2010). The genomes of this endophyte did not show significant sequence homology with the taxol biosynthetic genes from *Taxus* spp., which indicates that the fungus might have independently developed the taxol biosynthesis pathway (Heinig et al., 2013). The hypothesis by Howitz and Sinclair (2008) states that homologous gene clusters present in plants and microorganisms may get cross-activated by stress-induced molecules from plant hosts or endophytes under certain conditions.

There are several reports on the precise effect of endophytes on host secondary metabolites production, but the mechanisms involved are quite unknown. An endophytic actinobacterium *Pseudonocardia* induced artemisinin production in *Artemisia* plant by inducing the expression of cytochrome P_450_ monooxygenase and cytochrome P_450_ oxidoreductase genes (Li et al., 2012). In their study, Pandey et al. (2016a) found that endophytes (*Acinetobacter* sp. and *Marmoricola* sp.) of *Papaver somniferum* L. upregulate the expression of key genes for the biosynthesis of benzylisoquinoline alkaloid. Studies also indicate that *Catharanthus roseus*-bearing fungal endophytes *Curvularia* sp. and *Choanephora infundibulifera* induce expression of terpenoid indole alkaloid pathway genes (Pandey et al., 2016b; Sreekanth et al., 2017). In *Crotalaria*, biosynthesis of pyrrolizidine alkaloids (PAs), which is essential in plant’s chemical defense against herbivores, depends on the nodulation by *Bradyrhizobium* (Irmer et al., 2015). The presence of transcripts of homospermidine synthase (HSS), the first enzyme of the PA pathway, exclusively in the nodules, indicates that PA biosynthesis is restricted to the nodules. Therefore, the nodules are the source from which alkaloids are transported to the above-ground parts of the plant (Irmer et al., 2015). However, like PAs, there may be possibilities that many more bioactive secondary metabolites with novel applications could be synthesized by the presence of endophytes in the host plant.

## Conclusion and Perspectives

Endophytes represent an eco-friendly option for the promotion of plant growth and for serving as sustainable resources of novel bioactive natural products. Numerous endophytes and their genes have now been identified, which provide understanding about their behavior and mechanisms. Still, there are big gaps regarding the lifestyle and working of these microbes (Santoyo et al., 2016). Research shows that only about 1–2% of known plant species have been studied for endophytic associations (Strobel and Daisy, 2003) and most of these are the land plants, whereas aquatic plants (ocean, lakes, etc.,) are completely untouched (Strobel, 2018). It is high time to deduce the biochemistry and physiology of endophytes up to genomic and metabolomics levels. To date, there are no databases exclusively available for endophytic microorganisms and their metabolites, which can be of great importance and provide solutions to many issues. Several important bioactive compounds produced by endophyte-plant interactions can be utilized in various fields such as agriculture, medicine, bioremediation, and biodegradation (Table 1). Endophytes can also be employed as nanofactories for the synthesis of nanoparticles to reduce metals (Baker and Satish, 2012). The emerging use of endophytes-based nanoparticles has showed promising results for future drug development. In the near future, the application of endophytes may revolutionize drug formulations. Host plants can be induced to produce required metabolites of interest such as those used in drugs for treating cancer. Similarly, endophyte-harboring crop varieties, with induced resistance against phytopathogens, can also be designed. This could help definitely in the conservation of indigenous varieties as better alternatives to genetically engineered crops for maintaining the flavor and metabolite levels.

**Table 1:** Diverse roles played by endophytes in association with host plants.

Names of endophyte	Host-plant and site where reported	Reported roles	Reference
*Bacillus cereus* and *B. subtilis; Penicillium chrysogenum* and *P. crustosum.*	Leaves of *Teucrium polium*	PGP properties	Hassan, 2017
			
*Verruconis* strain SYPF 8337T	Root of *Panax notoginseng*	The first fungus isolated as an endophyte of *P. notoginseng*	Zhang et al., 2018
*Fusarium tricinctum* SYPF 7082	Root of *Panax notoginsen*g	Two new alkaloids, two known ones having therapeutic values	Sun W.J. et al., 2018
*Sphingomonas paucimobilis* and *Aspergillus* sp.	Leaves, stems and roots of *Lippia citriodora*	PGP properties	Golparyan et al., 2018
*Microspongium alariae*	Brown alga *Alaria esculenta*	Cell modification properties	Murúa et al., 2018
*Bacillus* spp. and *Pseudomonas* spp	Buds of *Malus domestica*	Apple shoot growth, cellular redox balance, and protein expression under *in- vitro* conditions	Tamošiûnë et al., 2018
*Aspergillus oryzae*	*Raphanus sativus*	PGP and protection against herbivore pest *Plutella xylostella*	Sun B.T. et al., 2018
*Enterobacter* sp. strain PDN3,	Cuttings of *Populus deltoides*xnigra	Endophyte-assisted phytoremediation of Trichloroethylene	Doty et al., 2017
*Pseudomonas stutzeri* A15	Rhizosphere and endosphere of *Oryza sativa*	PGP properties	Pham et al., 2017
*Serratia grimesi* BXF1 (Transformed strain for ACC deaminase activity)	*Pine pinaster*, *Solanum lycopersicum* and *Cucumis sativus*	Co-inoculation of *Serratia grimesii* BXF1 with *Rhizobium tropici* CIAT 899 promotes early nodulation and growth of common bean	Tavares et al., 2018
*Epichloë festucae* var. lolii	*Lolium perenne* grass	Bioactive alkaloids providing defense against herbivores	Fuchs et al., 2017
*Urnula* sp.	Stem of *Dicksonia antarctica*	Induced mixture of biologically active volatile organic compounds	Strobel et al., 2017
*Galactomyces geotrichum* WLL1	*Trapa japonica*	Enhance of nutrient uptake and modulate nutritional parameters	Waqas et al., 2017
*Exophiala pisciphila*	Root of *Zea mays*	Decrease in cadmium phytotoxicity and a significant increase in maize growth	Wang et al., 2016
*Penicillium canescens*	Roots, stems, and leaves of *Cajanus cajan*	Biotransformation of astragalosides to astragaloside IV	Yao et al., 2014
*Enterobacter* sp.	*Eleusine coracana*	Suppressing *Fusarium graminearum* in plant tissues and reduction of deoxynivalenol mycotoxin	Mousa et al., 2015
*Pseudomonas poae* RE^∗^1-1-14	Roots of *Beta vulgaris*	Production of novel lipopeptide Poaeamide suppressing *Phytophthora capsici* and *P. infestans* zoospores	Zachow et al., 2015


To harness the benefits, endophyte(s)-based bioformulations have to be developed in the future. Such bioformulations when applied on seeds or aerial parts will be far more effective because once the microbe is inside the plant tissue, it will not face the competition of other soil microbes, which is common in the case of rhizosphere microbes. Moreover, the benefits are directly transferred to the host in a closed-circuit system where leakage of metabolites is minimal. Bioformulations of endophytes can be particularly effective in conditions where abiotic stresses are prevalent. Endophyte-based bioformulations can also be very useful for remediation of contaminated soils. Endophytes may thus overcome many of the shortcomings associated with conventional bioformulations (Card et al., 2016). However, for being commercially successful as next-generation bioformulations, thorough understanding of the following are required to be researched upon: (i) genetic and molecular bases of plant-endophytes interactions, (ii) strategies to establish symbiotic association between endophyte and host plant, and (iii) mode of transmission (endophytes could be of interest in that they are also transmitted vertically through plant reproductive tissues to the next generation).

Endophytes are very important biological resources, which need to be explored in the future to achieve targets of environmental sustainability, to act as unlimited sources of biomolecules for different industrial sectors and to those related directly to human health. The need is to investigate genomics and the integrated metabolism of the plant-endophyte relationship in order to garner benefits from this remarkable association.

## Author Contributions

NKA and EK conceived the idea. NKA, EK, and JM prepared the manuscript. JM prepared illustration. NKA supervised the whole study.

## Conflict of Interest Statement

The authors declare that the research was conducted in the absence of any commercial or financial relationships that could be construed as a potential conflict of interest.
